# A cross‐sectional analysis of emotional and binge eating in UK adults enrolled on the NHS low‐calorie diet pilot for type 2 diabetes

**DOI:** 10.1111/cob.70025

**Published:** 2025-06-18

**Authors:** Jordan Marwood, Duncan Radley, Tamla Evans, Jamie Matu, Kenneth Clare, Chirag Bakhai, Louisa J. Ells

**Affiliations:** ^1^ Obesity Institute and School of Health Leeds Beckett University Leeds UK; ^2^ Obesity Institute and School of Sport Leeds Beckett University Leeds UK; ^3^ Larkside Practice, Churchfield Medical Centre Bedfordshire UK

**Keywords:** binge eating, diabetes remission, disordered eating, emotional eating, low‐calorie diet, total diet replacement, type 2 diabetes

## Abstract

This study presents data gathered as part of the Re:Mission evaluation of the NHS low‐calorie diet programme pilot for type 2 diabetes, to address two research questions: (1) What is the presence and severity of emotional and binge eating within this population? (2) Are demographic and health factors associated with the presence of binge eating or the severity of emotional eating? An online survey gathered data from *n* = 580 individuals who were enrolled on the programme but had not yet begun total diet replacement. The survey assessed emotional eating (TFEQ‐R21), potential binge eating disorder diagnosis (BEDS‐7), wellbeing (Short Warwick‐Edinburgh Mental Wellbeing Scale), quality of life (EQ‐5D‐5L), frequency of weight cycling and demographic factors (collected via NHS England programme monitoring). Descriptive statistics and regression analyses were used to address the research questions. The mean emotional eating score from the TFEQ‐R21 was 2.58 (0.96), and the presence of a potential binge eating disorder diagnosis was demonstrated in 26.0% of the sample. Regression analyses suggested that being female and engaging in more frequent weight cycling were associated with higher emotional eating and a greater likelihood of binge eating. Lower wellbeing and lower quality of life were associated with emotional and binge eating, respectively. Rates of emotional and binge eating in the NHS low‐calorie diet pilot sample are higher than in the general public and in other similar samples. Consideration should be given to screening for emotional and disordered eating and for additional tailored support and monitoring for such individuals.

**What is already known about this subject?**

- Previous studies have suggested that emotional and binge eating are prevalent in people living with obesity and in those with type 2 diabetes.
- The rates of emotional and binge eating in individuals accessing low‐calorie diet interventions are unknown.

**What this study adds**

- This study presents cross‐sectional data on the rates and severity of binge and emotional eating in a sample of UK adults accessing a low‐calorie diet programme for type 2 diabetes.
- The findings suggest that being female, engaging in more frequent weight cycling, and having a low wellbeing or poorer quality of life are associated with a greater risk for binge eating/higher emotional eating at baseline in this sample.

## 1 INTRODUCTION

Obesity and type 2 diabetes mellitus (T2DM) present significant and enduring risks to both individual^1^, ^2^ and public health,^3^, ^4^ as well as having economic implications for health systems worldwide.^5^, ^6^ These non‐communicable diseases have multiple comorbidities, including emotional and disordered eating, which can add further complexity to effective prevention and treatment. Emotional eating is described as the tendency to overeat in response to negative emotions^7^ and is associated with poorer weight outcomes longitudinally and in response to lifestyle and behavioural interventions.^8^ Although rates of emotional eating are difficult to quantify due to the range of measures used to assess this construct, evidence thus far suggests prevalence ranges from 20.5% in a nationally representative US community sample^7^ to 58% in a sample of Australian adults attending specialist weight management treatment.^9^ These rates are problematic, as a recent systematic review found emotional eating to be associated with overweight, obesity, depressive symptoms, and psychological distress.^10^

Disordered eating, specifically binge eating, is also associated with the heightened negative emotions that may precede emotional eating, but presents in a more clinically recognisable and severe pattern of behaviour.^11^ Binge eating disorder (BED), an eating disorder in which binge eating is the main symptom, is the most common eating disorder worldwide^12^ and is strongly associated with lifetime prevalence of obesity, with research suggesting rates of obesity are as high as 87% in an inpatient sample with BED.^13^ This may be because BED is not associated with inappropriate compensatory behaviour such as purging, which is seen in Bulimia Nervosa. Furthermore, the rates of BED in people with T2DM are higher than the general population, with a systematic review finding rates range from 1.2% to 25%,^12^, ^14^ with some evidence that those with comorbid BED and T2DM may have higher body mass index (BMI) than those without BED.^14^ Importantly, evidence suggests that in those with both conditions, the disordered eating behaviour predates T2DM,^15^ indicating a potential causal effect. Despite clear associations between T2DM, obesity, emotional and binge eating, there is a lack of evidence exploring their relationship,^16^ and the role that demographic and health variables may have in their likelihood or severity.

Recent evidence suggests that the implementation of a low‐calorie diet (LCD) may have sustained positive impacts on both BMI and blood glucose control^17^, ^18^, ^19^ in people with T2DM; however, the prevalence and severity of emotional and binge eating in people accessing these services in the UK are unknown. Although people with a known eating disorder are excluded from LCD participation, given the high rates of disordered eating in people with T2DM, it is likely that many participants are living with disordered eating, and evidence suggests that BED often goes undiagnosed.^12^ Addressing emotional eating may be crucial in supporting effective weight loss,^20^ and whilst for most people weight management interventions do not impact disordered eating, there is a subset of individuals for whom such interventions may have a deleterious effect on their eating behaviour.^21^ Better understanding the factors that are associated with disordered and emotional eating in people accessing these interventions is therefore a priority.

The current study presents data from a sample of UK adults who accepted a referral to a low‐calorie intervention for T2DM to answer the following research questions: (1) What is the presence and severity of emotional and binge eating within this population? (2) Are demographic and health factors associated with the presence of binge eating or the severity of emotional eating?

## 2 METHODS

### 2.1 Design

This study reports data gathered as part of a participant survey collected for the Re:Mission evaluation (NIHR132075) of the National Health Service (NHS) LCD programme pilot. The programme consisted of three phases: total diet replacement (TDR) lasting 12 weeks, food reintroduction (4–6 weeks) and weight maintenance until the end of the programme (52 weeks). Different delivery models were included in the pilot, including one‐to‐one, group, and digital delivery through an app. The data presented here was collected at baseline, before participants began TDR.

### 2.2 Participants

Participants were all individuals enrolled on the LCD programme, who had not yet begun the TDR phase. To be eligible for the programme, participants needed to have received a diagnosis of T2DM within the last 6 years, be non‐insulin dependent, and have a BMI ≥27 kg/m^2^ (or ≥25 kg/m^2^ for Black, Asian and minority ethnic communities). A total of *n* = 719 participants completed the survey; however, a proportion of these did not provide the appropriate referral ID; therefore, it was not possible to link their survey data to the NHS England (NHSE) dataset. As the NHSE dataset includes important demographic data (detailed below), the decision was made to exclude these participants due to lack of data linkage, leaving a total *n* = 580.

### 2.3 Procedure

Data was collected using surveys that were co‐developed by NHSE, Diabetes UK, service providers, service users, and the study Patient and Public Involvement and Engagement group. Surveys were completed anonymously online via Qualtrics (Provo, UT) software, and service users were invited to participate by an email sent to them by their service provider between September 2021 and April 2023. The content of the survey was presented in two parts: (1) experiences of the programme; (2) lifestyle, physical health, and wellbeing. This paper reports on data collected in the second part of the survey, in addition to sociodemographic data collected by NHSE as part of LCD programme monitoring.

### 2.4 Survey measures

#### 2.4.1 Three factor eating questionnaire‐R21: emotional eating subscale

The three factor eating questionnaire‐R21: emotional eating subscale (TFEQ‐R21 EE) comprises six items and is scored on a Likert scale ranging from 1 to 4. Domain scores are calculated as the mean of items within each domain, and higher scores indicate higher levels of emotional eating.

#### 2.4.2 Binge eating disorder screener‐7

This patient‐reported screener is used to assess whether a patient may have BED. If the patient answers in the affirmative to both question 1 and question 2 (assessing presence of and distress about binge eating), answers “sometimes”, “often” or “always” in response to questions about other binge eating symptoms (i.e. loss of control, guilt) and does not endorse vomiting as a means of weight or shape control then they are flagged as “potential BED diagnosis” for the purposes of this analysis.

#### 2.4.3 Short Warwick‐Edinburgh Mental Wellbeing Scale

This measure of wellbeing includes seven items each scored on a Likert scale ranging from 1 to 5. Items are summed, and raw scores are then transformed into metric scores.^22^ Scores range from 7 to 35, and higher scores indicate greater mental wellbeing.

#### 2.4.4 EQ‐5D‐5L

This measure of quality of life asks participants the difficulties that they have (ranging from no problems to extreme problems) on five dimensions of quality of life: mobility, self‐care, usual activities, pain/discomfort and anxiety/depression. Following NICE guidance,^23^ the raw scores were “crosswalked” into the EQ‐5D‐3L scoring system using a mapping function developed by van Hout et al.^24^ This creates an index score with a possible maximum of 1.

#### 2.4.5 Frequency of weight cycling

Participants were asked to state the times over their life (prior to starting the programme) that they lost more than 11 lbs/5 kg in weight by dieting, with the response options of never, 1–2 times, 3–5 times, and over 5 times coded numerically from 1 to 4 to demonstrate increasing frequency of weight cycling.

### 2.5 Minimum dataset variables

Data from the survey was anonymously linked to data collected by NHSE as part of their minimum dataset. These variables included sex, ethnicity, age, BMI, duration of diabetes and baseline HbA1c.

### 2.6 Analysis

Independent sample t‐tests and chi square were conducted to determine if there were any statistical differences in mental wellbeing (Short Warwick‐Edinburgh Mental Wellbeing Scale (SWEMWS)), quality of life (EQ‐5D‐5L), emotional eating score (TFEQ‐R21) and BED‐7 binge screening (yes/no to likely BED) between the scores of the participants excluded (from the original 719) and those included (*n* = 580).

To address research question 1 descriptive statistics are presented for all variables of interest. To address research question 2, two regression analyses were conducted; the first was a linear regression, with sex, ethnicity (for the purpose of the analysis, ethnicity was coded as ‘White’ or ‘Global Majority’), BMI, duration of diabetes, HbA1c, frequency of weight cycling, mental wellbeing (SWEMWS) quality of life (EQ‐5D‐5L) as the predictor variables and TFEQ‐R21 emotional eating score as the outcome variable. The second model was a logistic regression with the same predictor variables and BED‐7 binge screening (yes/no to likely BED) as the outcome variable. The assumptions of both modes of analysis were examined and met,^25^ and the alpha level was set at <.05.

### 2.7 Ethics

Ethical approval was received from Leeds Beckett University [ref: 107887] and written informed consent was obtained from participants.

## 3 RESULTS

### 3.1 Descriptive statistics

Demographic, health, and provider data can be seen in Table 1. The majority of the sample was female and of White ethnicity. The sample ranged from 18 to 65 years of age and the mean age of participants was 50.3 (9.6) years. The mean BMI was 38.3 (7.0) kg/m^2^, ranging from 25.3 to 67.1 kg/m^2^. Baseline HbA1c ranged from 43.0 to 87.0 mmol/mol/6.1%–10.1% with a mean of 58.2 (10.6) mmol/mol/7.5 (1.0)%. Duration of diabetes diagnosis ranged from 13 to 2369 days, with an average of 714 days (approximately 23 months). Most service users were enrolled in the group delivery model, and just under half of the sample were having the programme delivered by provider 2 (provider names removed for anonymity).

**TABLE 1:** Demographic, health, and provider data.

Variable	Frequency/mean (percentage/SD)
Sex	
Male	225 (38.8%)
Female	355 (61.2%)
Ethnicity	
White	467 (80.5%)
Mixed	15 (2.6%)
Asian or Asian British	48 (8.3%)
Black or Black British	38 (6.6%)
Other ethnic group	5 (0.9%)
Not stated	7 (1.2%)
Age (years)	
<30	16 (2.8%)
30–<40	70 (12.1%)
40–<50	157 (27.1%)
50–<60	228 (39.3%)
60+	109 (18.8%)
BMI (kg/m^2^)	38.3 (7.0)
HbA1c (mmol/mol)	58.2 (10.6)
HbA1c (%)	7.5 (1.0)
Duration of diabetes (days)	714 (668)
Frequency of weight cycling	
Never	133 (22.9%)
1–2 times	250 (43.1%)
3–5 times	117 (20.2%)
>5 times	80 (13.8%)
Delivery model	
1‐2‐1	147 (25.3%)
Group	346 (59.7%)
Digital	87 (15.0%)
Provider^a^	
1	35 (6.0%)
2	271 (46.7%)
3	144 (24.8%)
4	51 (8.8%)
5	57 (9.8%)
6	22 (3.8)

*Note*: *N* = 580.

Abbreviations: BMI, body mass index; SD, standard deviation.

^a^ Provider names removed for anonymity.

Table 2 displays the descriptive statistics from measures of emotional eating, binge eating, mental wellbeing, and quality of life. Just over a quarter of the sample screened positive on the binge eating disorder screener‐7 (BEDS‐7) for likely BED. The emotional eating subscale of the TFEQ‐R21 ranged from 1 to 4, with a mean of 2.58. The SWEMWS data indicated mental wellbeing scores that reflect UK population norms.^26^ The EQ‐5D‐5L ranged from −0.33 to 1, with a mean score of 0.73, which suggests slightly lower quality of life than UK population norms.^27^

**TABLE 2:** Eating behaviour, mental wellbeing and quality of life.

Variable	Frequency/mean (percentage/SD)
TFEQ‐R21‐EE (*n* = 540)	2.58 (0.96)
BEDS‐7 (*n* = 543)	
Yes	141 (26.0%)
No	402 (74.0%)
SWEMWS (*n* = 549)	23.5 (4.7)
EQ‐5D‐5L (*n* = 549)	0.73 (0.27)

Abbreviations: BEDS‐7, binge eating disorder screener‐7; SWEMWS, Short Warwick Edinburgh Mental Wellbeing Scale; TFEQ‐R21‐EE, three factor eating questionnaire‐R21: emotional eating subscale.

There were no significant differences between the scores of the participants excluded (from the original 719) and those included (*n* = 580) for the emotional eating subscale of the TFEQ‐R21 (2.58 vs. 2.62, *p* = .65), the EQ‐5D‐5L (0.73 vs. 0.74, *p* = .74), SWEMWS (23.5 vs. 23.3, *p* = .64) and the proportion screened positive on the BEDS‐7 for likely BED (26.0% vs. 21.2%, *p* = .28).

### 3.2 Linear regression

As can be seen in Table 3, the linear regression model was significant (*p* < .001), accounting for approximately 21% of the variance. Examination of the coefficients suggests that participant sex (*p* < .001), frequency of weight cycling (*p* = .02) and SWEMWS score (*p* < .001) all significantly contributed to the model. The direction of the effects shows that being female, greater frequency of weight cycling, and lower wellbeing were associated with a greater emotional eating score on the TFEQ‐R21.

**TABLE 3:** Linear regression model.

Variable	Overall model (*n* = 526)				
	*B*	SE	Beta	*t*	*p*
Sex	−.420	.429	−.215	−5.44	**<.001**
Age	−.002	.077	−.051	−1.20	.232
BMI	.010	.004	.071	1.68	.093
Ethnicity	.181	.006	.072	1.75	.081
HbA1c	.000	.104	.001	.030	.976
Duration of diabetes (days)	.029	.004	.002	.039	.969
Frequency of weight cycling	.093	.000	.093	2.28	.**023**
EQ‐5D‐5L	−.087	.041	−.024	−.559	.576
SWEMWS	−.067	.155	−.325	−7.55	**<.001**
*R* ^2^	.479				
Adjusted *R* ^*2*^	.216				

*Note*: *F*(9, 516) = 17.09, *p* < .001. Emboldened values have an alpha of <.05.

Abbreviations: BMI, body mass index; SWEMWS, Short Warwick Edinburgh Mental Wellbeing Scale.

### 3.3 Logistic regression

Table 4 demonstrates that the model was significant (*p* < .001), accounting for approximately 11% of the variance. The coefficients suggest that participant sex (*p* = .004), frequency of weight cycling (*p* = .015), and quality of life (*p* = .011) all significantly contributed to the model. The direction of the effects demonstrate that being female, greater frequency of weight cycling, and lower quality of life were associated with a higher likelihood of being flagged as “potential BED diagnosis” on the BEDS‐7.

**TABLE 4:** Logistic regression model.

Variable	Overall model (*n* = 529)				
	*B*	SE	Wald's *χ* ^2^	*p*	Odds ratio
Sex	−.639	.224	8.09	.**004**	.528
Age	−.015	.012	1.62	.203	.985
BMI	.003	.015	.031	.859	1.00
Ethnicity	.356	.318	1.25	.263	1.43
HbA1c	.001	.010	.006	.937	1.00
Duration of diabetes (days)	.000	.000	.754	.385	1.00
Frequency of weight cycling	.273	.112	5.90	.**015**	1.31
EQ‐5D‐5L	−1.03	.405	6.43	.**011**	.358
SWEMWS	−.044	.026	2.91	.088	.957
*χ* ^2^	43.36				
Nagelkerke's pseudo *R* ^*2*^	.115				

*Note*: Emboldened values have an alpha of <.05.

Abbreviations: BMI, body mass index; SWEMWS, Short Warwick Edinburgh Mental Wellbeing Scale.

## 4 DISCUSSION

This study sought to examine the presence and severity of emotional and binge eating in a cohort of participants enrolled on the NHS LCD programme for T2DM, as well as to determine whether any health or sociodemographic factors were associated with emotional or binge eating in this sample.

The mean emotional eating score from the TFEQ‐R21 was 2.58 in this sample. This is higher than rates reported in a Taiwanese sample (*M* = 1.90) of people with overweight,^28^ and is also slightly higher than a similar American sample (*M* = 2.31) of people living with obesity.^29^ Although these samples were people living with overweight/obesity, they were either not assessed for diabetes^28^ or only a small percentage of the sample had a diabetes diagnosis.^29^ These results could therefore suggest that emotional eating is more severe in those with comorbid T2DM and obesity, or that emotional eating is higher in UK samples. However, the heterogeneity of measures used to assess emotional eating presents a problem for comparison with other samples, and greater consistency in assessment is needed.

When examining factors associated with emotional eating, it was found that being female, frequent weight cycling, and lower wellbeing were all associated with higher mean scores on the emotional eating subscale of the TFEQ‐R21. These findings align with previous research which demonstrates that emotional eating is higher in women^30^ and that higher levels of emotional eating are reported by individuals who are “weight unstable” relative to those with a stable weight.^31^ The current findings provide new evidence on the importance of these factors in people with obesity and T2DM. The role of wellbeing in emotional eating is complex, but emotional eating has been associated with depression and psychological distress.^10^ This is unsurprising when considering the theoretical underpinnings of emotional eating, which suggest this behaviour is a way to escape psychological distress or suppress emotions,^32^ which have been supported by empirical evidence.^33^ Indeed, incidents of emotional eating were found to increase substantially during the COVID‐19 pandemic, when wellbeing levels were low.^34^ The finding that lower wellbeing is associated with emotional eating therefore aligns with theoretical explanations and suggests that improving wellbeing may be a way to alleviate emotional eating. Previous literature is equivocal on the association between BMI and emotional eating, with some suggesting emotional eating is associated with overweight and obesity^10^ whilst other studies find a minimal association between these factors.^29^ Whilst it is important to note that the current findings are cross‐sectional and cannot give insight into the association between BMI and emotional eating or binge eating over time in those with T2DM and obesity, our finding that HbA1c or BMI are not predictive of emotional eating or the potential presence of BED is noteworthy. Given that most T2DM studies focus on HbA1c and most studies in people with obesity focus on weight and BMI, consideration of a wider set of variables related to emotional and disordered eating should be included in order to gain a more comprehensive understanding of participants' wellbeing.

The presence of potential BED was seen in 26.0% of the sample. The BEDS‐7 demonstrates a high level of sensitivity and reasonable specificity,^35^ suggesting that this figure is a reliable reflection of clinically severe binge eating in the sample. Previous research suggests that 15%–20% of people seeking weight loss have BED,^36^ this statistic was generated when the criteria for BED were more stringent, and rates are likely to be marginally higher using updated diagnostic criteria.^37^ The prevalence of potential BED diagnosis seen in the current sample is over double the rate reported in the Look AHEAD trial,^38^ which reported 11% incidence of binge eating in the past 6 months at baseline. Overall, the rates of potential BED diagnosis seen in this sample are much higher than those in the general population and in similar trials.

For binge eating behaviour, being female, frequent weight cycling, and poorer quality of life were associated with the likelihood of being flagged with a probable BED diagnosis. Previous studies have demonstrated an association between higher frequency of weight cycling and greater prevalence of binge eating in people with obesity,^39^ and binge eating is well‐established to be more common in women than men,^36^ suggesting that the current findings align with the literature. Poorer quality of life in people who binge eat has been reported in clinical samples of people with BED^40^, ^41^ and the current findings extend this to demonstrate the association between these variables in a sample of people with obesity and T2DM. Although these findings cannot demonstrate causality, they do suggest a clear relationship between binge eating and poorer quality of life in this sample.

Overall, this study demonstrates that rates of emotional and binge eating in the LCD sample are higher than in the general public and in other similar samples. Despite individuals with known eating disorders being ineligible for LCD participation, a quarter of participants met criteria for potential clinical diagnosis at baseline. The results also suggest that being female, greater frequency of weight cycling, poorer quality of life, and poorer wellbeing are associated with emotional and binge eating. These findings are pertinent to public health professionals who design, commission, and implement weight management interventions for T2DM. They demonstrate that there is a substantial cohort of individuals who are accessing routinely commissioned care, for whom highly restrictive diets may not be appropriate, particularly when disordered eating behaviour is not being monitored.^42^ This may ultimately result in higher demand on specialist services or may contribute to weight management programmes being ineffective for some individuals who experience common forms of disordered eating. Additionally, session observation data from the Re:Mission evaluation indicates limited support for emotional and disordered eating, which is corroborated by interview data from service users.^43^ These findings may also highlight issues within the health system in detecting and diagnosing eating disorders in people at a higher weight, potentially pointing to a lack of understanding of the range of eating disorders.

These findings are particularly important given that people with an eating disorder are more likely to seek support for their weight than they are for their eating behaviour^44^ and that less than half of people with BED will ever receive treatment or support, largely due to a considerable gap in understanding among healthcare professionals.^12^ Indeed, women with T2DM and BED report that a lack of knowledge about BED among healthcare providers resulted in unhelpful treatment, diminished trust, and feelings of guilt.^45^ This paper echoes the recommendations of others^46^, ^47^ that there is an important need for the development of protocols for the standardised assessment and appropriate management of disordered eating in people at a higher weight across all weight management interventions.

## 5 STRENGTHS AND LIMITATIONS

This study provides valuable insight into the prevalence of emotional and binge eating in a sample of people living with obesity and T2DM who are accessing a total diet replacement‐based weight management programme (NHS‐LCD). However, this study is limited by its cross‐sectional nature; although the survey was conducted at multiple time points, the response rate was not sufficient to enable longitudinal analysis; therefore, only baseline data is reported. Therefore, we still do not know the impact of the LCD programme on disordered and emotional eating in the short or long term, nor the extent to which disordered and emotional eating impact success and outcomes, and whether this differs by service user characteristics. It is therefore vital to further explore this issue and ensure that interventions are having the intended impact.

## 6 RECOMMENDATIONS

- Consideration should be given to screening for emotional or disordered eating in people entering weight management programmes. There are a range of appropriate tools to do this in people with T2DM.^12^
- People who screen positively for emotional or disordered eating may need additional tailored support and monitoring. Further research is required to develop the content and delivery of this support, which will require greater integration of disordered eating, obesity and diabetes support,^48^, ^49^ and should build on emerging areas of promising practice.^50^, ^51^
- There is a need for studies to assess the longer‐term implications for LCD interventions on disordered eating behaviour,^21^ and whether disordered and emotional eating varies by sociodemographic factors, to better understand cultural implications.

## CONFLICT OF INTEREST STATEMENT

All authors confirm that they have no conflicts of interest to declare. Louisa Ells has received funding from NIHR, MRC, Leeds City Council, and OHID/PHE in the last 3 years and has had an honorary contract with OHID. KC is a member of the Patient Advisory Board at Boehringer Ingelheim.
